# Supplementary figures and images for: The vaccinia chondroitin sulfate binding protein drives host membrane curvature to facilitate fusion
Source: EMBO Rep. 2024 Feb 6;25(3):1310–25. doi: 10.1038/s44319-023-00040-2 (PMC10933376; doi:10.1038/s44319-023-00040-2)

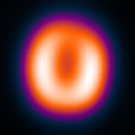

Supplement: Supplementary file 2 — Source Data Fig. 2 [file 44319_2023_40_MOESM2_ESM.zip › Fig. 2/2A/H3 model.png]

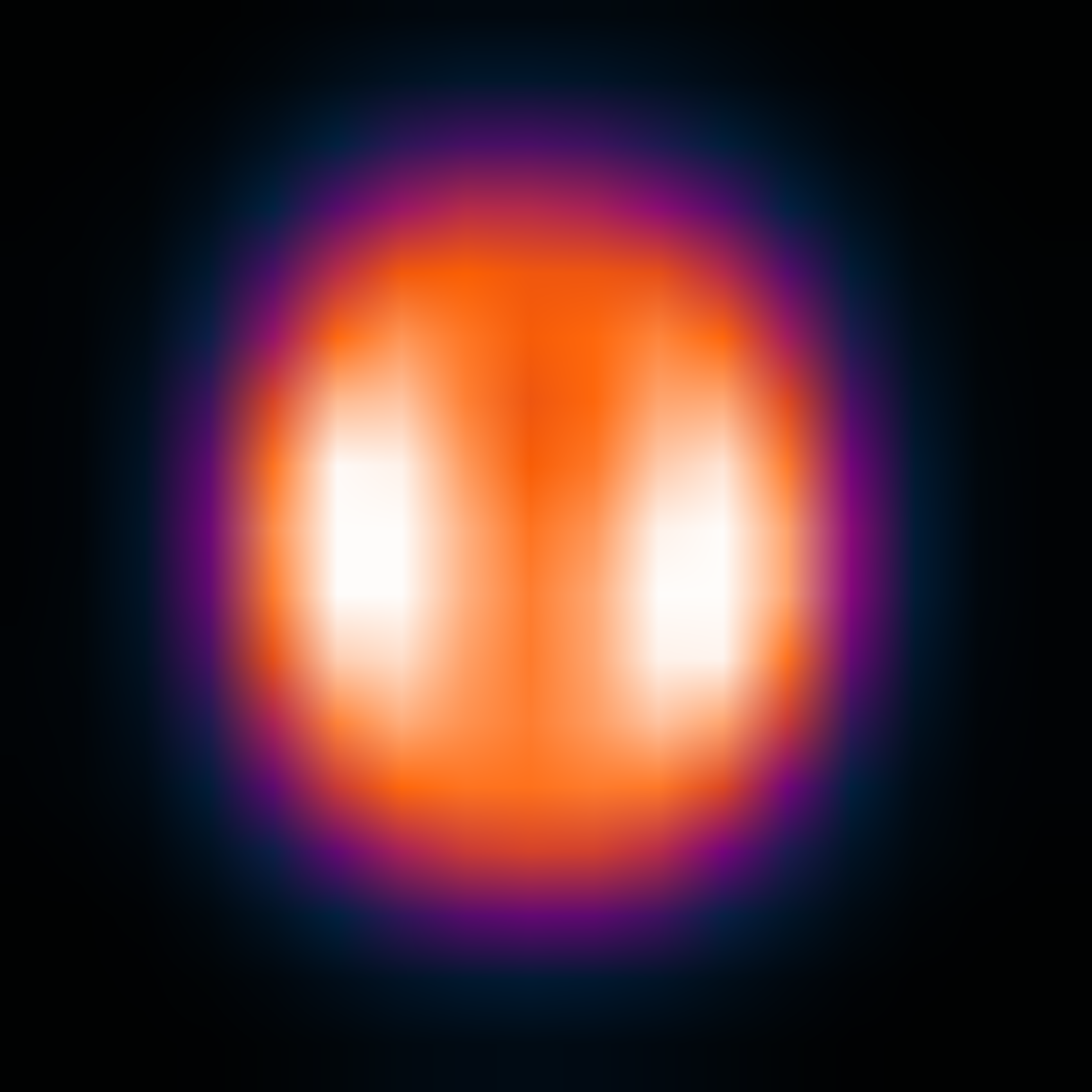

Supplement: Supplementary file 2 — Source Data Fig. 2 [file 44319_2023_40_MOESM2_ESM.zip › Fig. 2/2A/A26 model.png]

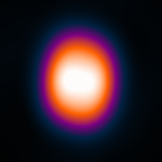

Supplement: Supplementary file 2 — Source Data Fig. 2 [file 44319_2023_40_MOESM2_ESM.zip › Fig. 2/2A/Core model.png]

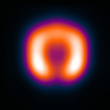

Supplement: Supplementary file 2 — Source Data Fig. 2 [file 44319_2023_40_MOESM2_ESM.zip › Fig. 2/2A/D8 model.tif]

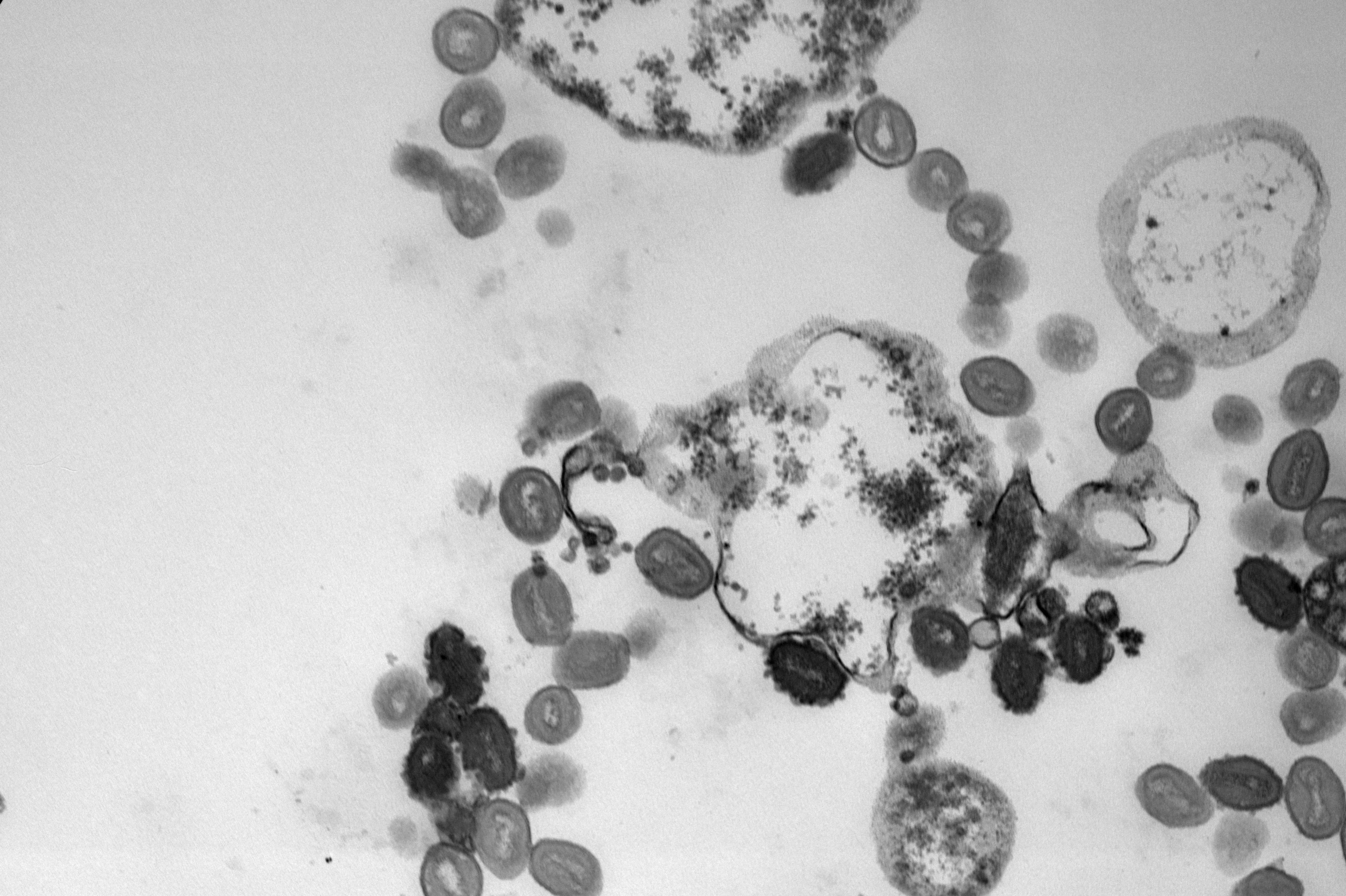

Supplement: Supplementary file 2 — Source Data Fig. 2 [file 44319_2023_40_MOESM2_ESM.zip › Fig. 2/2C/Fig. 2C WT .TIF]

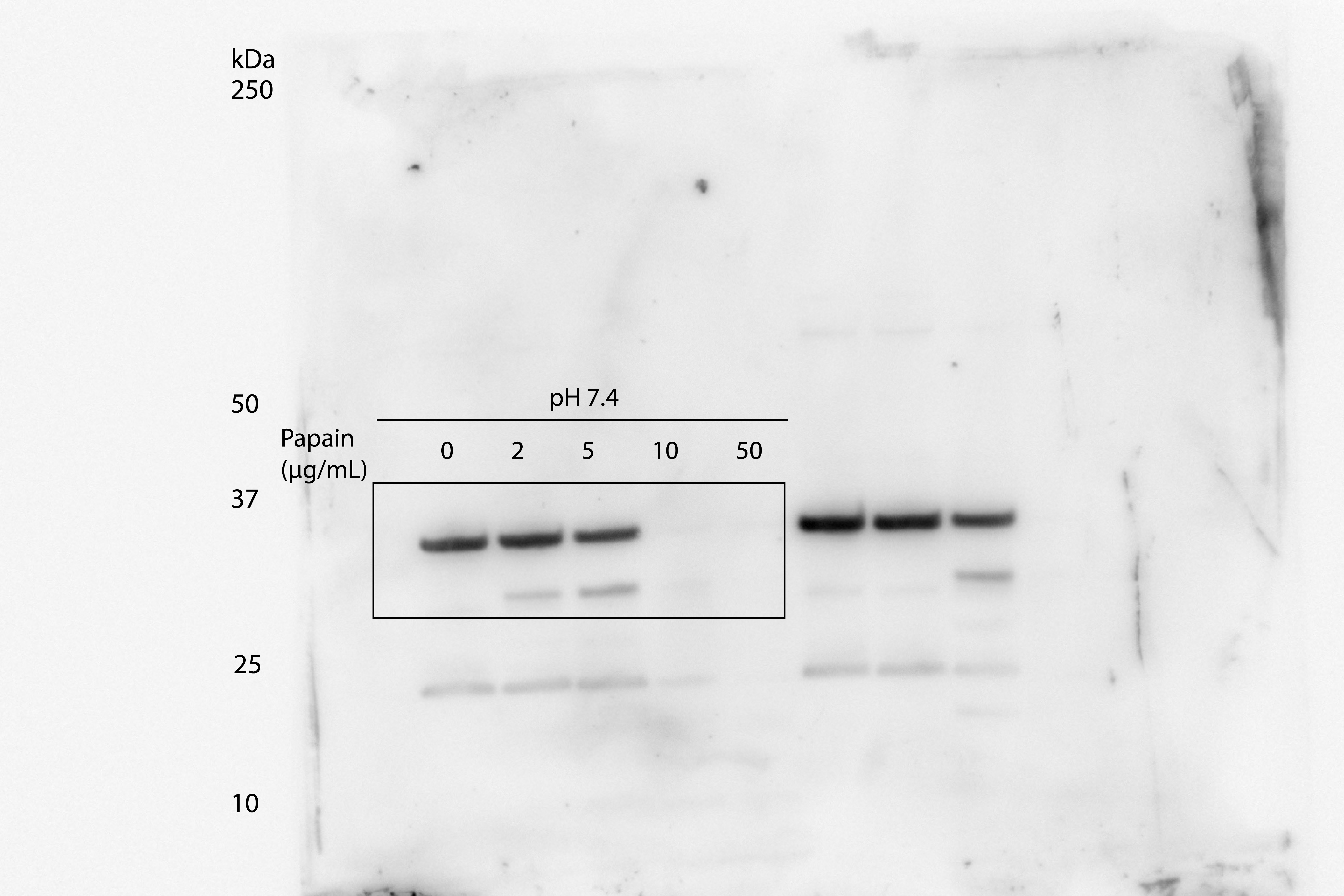

Supplement: Supplementary file 4 — Source Data Fig. 4 [file 44319_2023_40_MOESM4_ESM.zip › Fig. 4/4I/Fig. 4i_PapainDigest1.png]

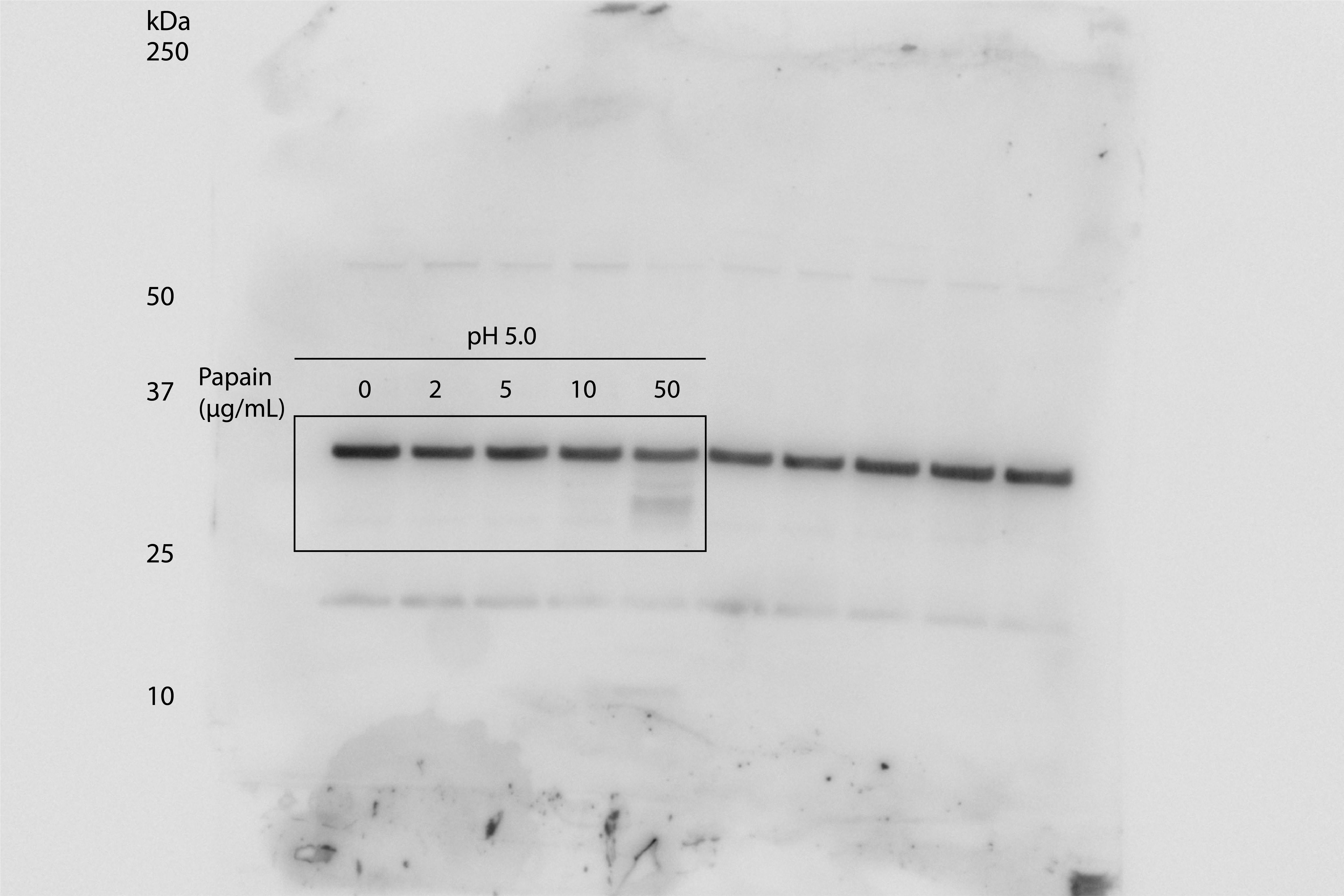

Supplement: Supplementary file 4 — Source Data Fig. 4 [file 44319_2023_40_MOESM4_ESM.zip › Fig. 4/4I/Fig. 4i_PapainDigest2.png]

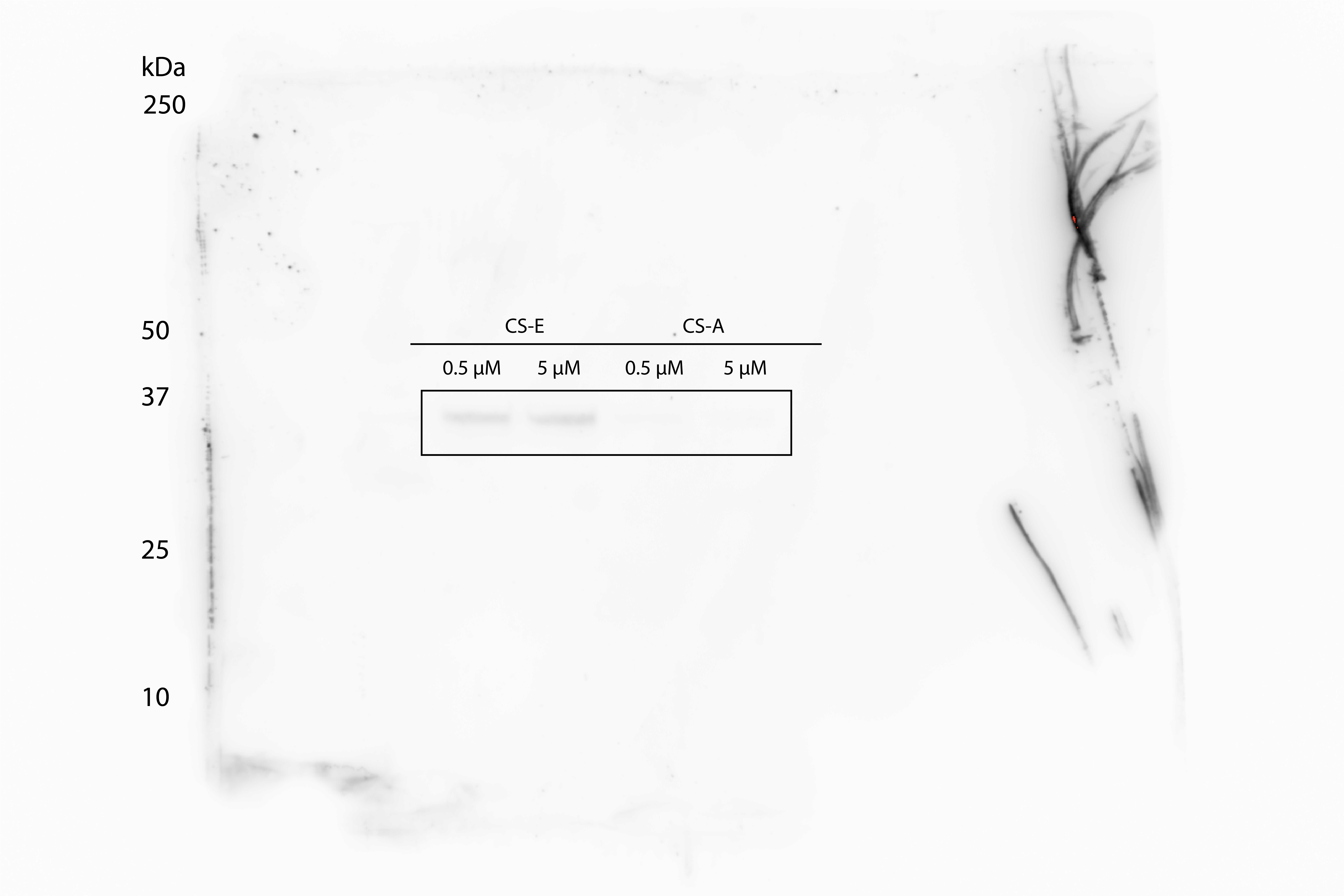

Supplement: Supplementary file 4 — Source Data Fig. 4 [file 44319_2023_40_MOESM4_ESM.zip › Fig. 4/4G/Fig. 4g_CSEorCSApulldownwithD8.png]
