# Supplementary material for: The vaccinia chondroitin sulfate binding protein drives host membrane curvature to facilitate fusion
Source: EMBO Rep. 2024 Feb 6;25(3):1310–25. doi: 10.1038/s44319-023-00040-2 (PMC10933376; doi:10.1038/s44319-023-00040-2)
Supplement: Supplementary file 6 — Expanded View Figures [file 44319_2023_40_MOESM6_ESM.pdf]

Expanded View Figure

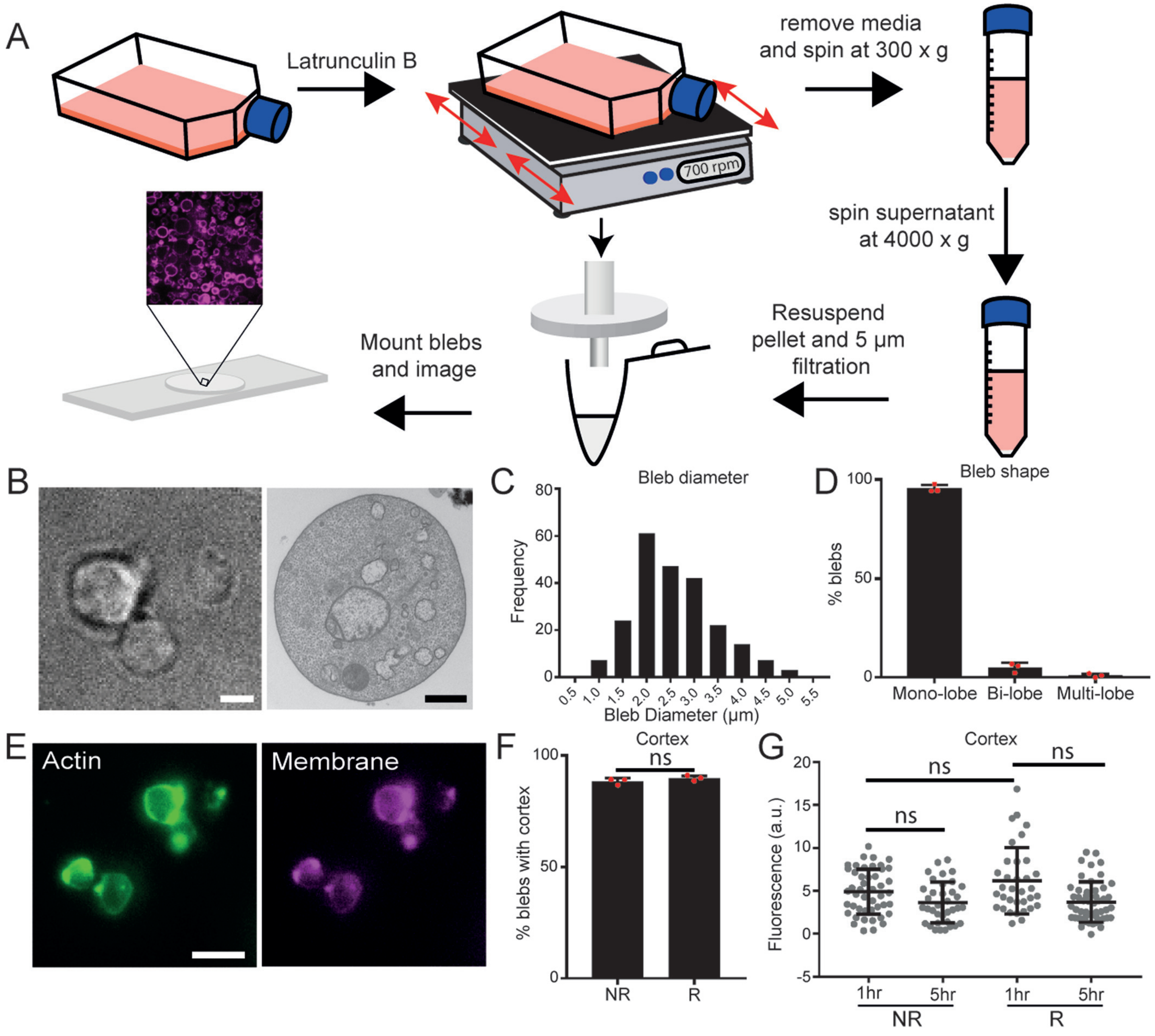

Figure EV1. Cell-derived membrane blebs as a minimal cell system.

(**A**) To prepare blebs Latrunculin B is added to cells to induce blebbing. Cells are shaken to detach blebs, cellular debris removed by a slow spin step (300 × *g*), blebs collected by a fast spin step (4000 × *g*), and remaining large debris removed by filtration through a 5 µm pore filter. (**B**) Representative brightfield (LHS, scale bar; 1 µm) and TEM (RHS, scale bar; 500 nm) images of blebs after purification. (**C**) Histogram of bleb diameter range. (**D**) Blebs were scored for mono-, bi- and multi-lobulation ($n > 100$ blebs/repeat). (**E**) Blebs were stained for actin (green) and the plasma membrane (PM; magenta). Scale bar, 5 µm. (**F**) Bleb cortical actin was reconstituted (R) with ATP or not (NR), stained for both actin and the PM and the percentage of blebs with an actin cortex calculated ($n > 100$ blebs/repeat). (**G**) Stability of the actin cortex over time at 37 °C between NR and R blebs was determined by intensity measurements of the actin stain on *z*-projections ($n > 40$ blebs). Data information: For (**D**, **F**, **G**), data are means ± SD. Statistical analysis was performed using unpaired two-tailed *t* tests (ns, not significant ($P > 0.05$)).

 © The Author(s)

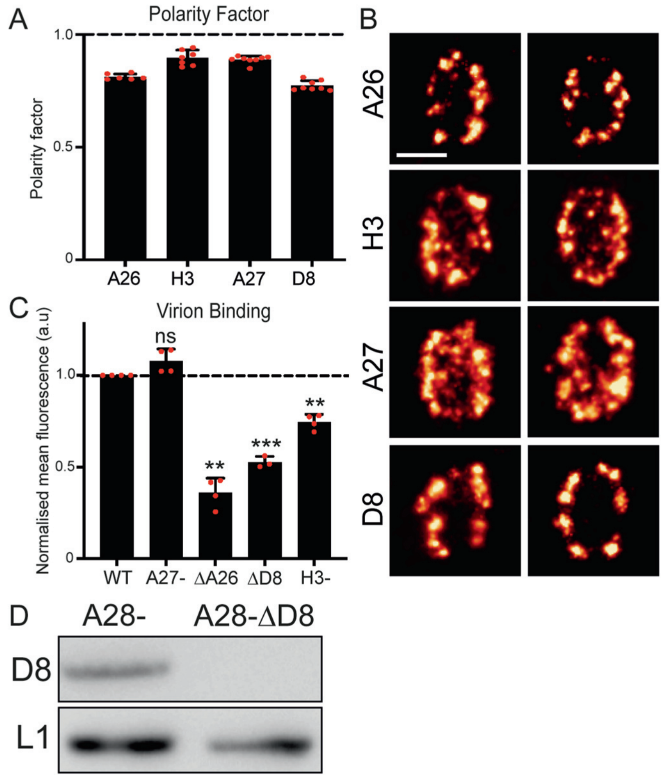

Figure EV2. VACV binding proteins reside at virion sides and are differentially required for VACV binding.

(**A**) Quantification of VACV binding protein polarity factors using data from the models in Fig. 2A. A polarity factor of less than one corresponds to concentration of the protein at the sides of MVs ($n = 50$ virions per repeat). (**B**) Representative STORM images of VACV binding proteins on individual MVs. Scale bar = 200 nm. (**C**) Binding affinities of WT and recombinant binding protein mutant VACVs on HS + CS+ cells ($n = 4$ biological repeats). (**D**) Representative immunoblot of D8 protein packaging in A28- and A28-ΔD8 virions. Molecular weight markers are indicated at right. Data information: (**A**, **C**) data are means ± SD. Statistical analysis was performed using unpaired two-tailed t tests (***$P < 0.001$; **$P < 0.01$; ns, not significant ($P > 0.05$)).

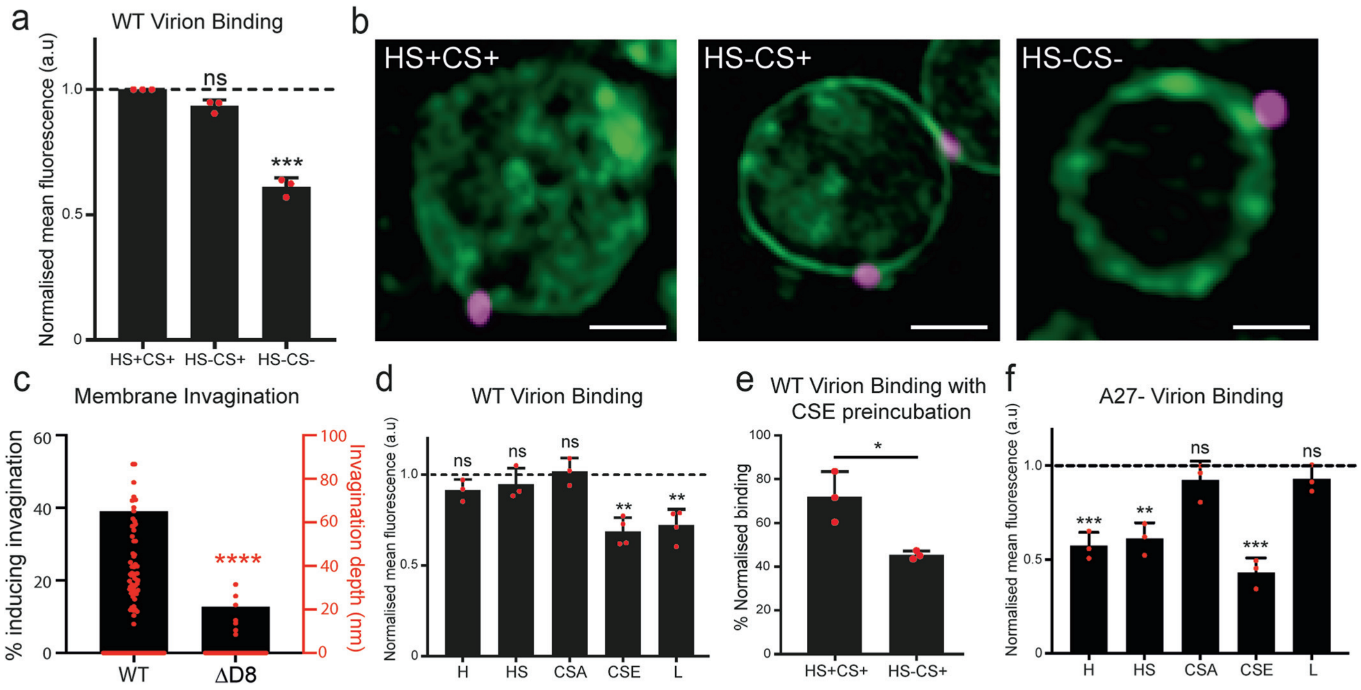

Figure EV3. CS-E is the major GAG used by VACV for binding.

(**A**) Binding affinities of WT VACV on HS + CS +, HS-CS+ and HS-CS- cells. (**B**) SIM images of VACV bound to HS + CS +, HS-CS+ and HS-CS- derived blebs scale bar = 2 µm. A4-EGFP VACV (magenta) and PM (green). (**C**) Quantification of % invagination and invagination depth of WT and ΔD8 virions on HS + CS+ cells ($n > 60$ virions/mutant). (**D**) Binding affinities of WT VACV with GAG pre-incubation on HS + CS+ cells. (**E**) Binding affinities of WT virus with CS-E preincubation on HS + CS+ and HS-CS+ cells. Data is normalized to no CS-E preincubation on the given cell type. (**F**) Binding affinities of A27- virions with GAG pre-incubation on HS + CS+ cells. Data information: (**A**, **D**–**F**) data are means ± SD. Statistical analysis was performed using unpaired two-tailed *t* tests (***$P < 0.001$; **$P < 0.01$; *$P < 0.05$; ns, not significant ($P > 0.05$)).

 © The Author(s)
